# Improved survival outcomes and relative youthfulness of multiple myeloma patients with t(4;14) receiving novel agents are associated with poorer performance of the revised international staging system in a real aging society

**DOI:** 10.18632/oncotarget.26562

**Published:** 2019-01-15

**Authors:** Yoshiaki Abe, Kazutaka Sunami, Takeshi Yamashita, Mikio Ueda, Hiroyuki Takamatsu, Kentaro Narita, Hiroki Kobayashi, Akihiro Kitadate, Masami Takeuchi, Kosei Matsue

**Affiliations:** ^1^ Division of Hematology/Oncology, Department of Internal Medicine, Kameda Medical Center, Chiba, Japan; ^2^ Department of Hematology, National Hospital Organization Okayama Medical Center, Okayama, Japan; ^3^ Department of Internal Medicine, Keiju Kanazawa Hospital, Ishikawa, Japan; ^4^ Department of Hematology/Respiratory Medicine, Faculty of Medicine, Institute of Medical, Pharmaceutical and Health Sciences, Kanazawa University, Ishikawa, Japan

**Keywords:** aging, multiple myeloma, prognosis, revised international staging system, t(4;14)

## Abstract

The Revised International Staging System (R-ISS) was developed for a more accurate risk stratification of patients with symptomatic multiple myeloma (MM). However, original and subsequent validation studies of the R-ISS included relatively younger patients, many of whom were treated without bortezomib. Hence, we investigated the real-world prognostic performance of the R-ISS in 400 patients with MM treated with novel agents in Japan, an aging society. The patients had a median age of 72 years, and 96.0% were treated with bortezomib. Patients in R-ISS stage II were significantly older and failed to show significantly longer overall survival (OS) compared to patients in R-ISS stages III (median age; 74 and 70 years, respectively; *P* = 0.001, and median OS; 63.4 vs. 54.7 months, respectively; *P* = 0.32). However, OS differed significantly among patients with all conventional ISS stages. ISS stage III patients recategorized to R-ISS stage III were significantly younger than those recategorized to R-ISS stage II and had a relatively longer OS. As a reason for these findings, patients with the high-risk cytogenetic abnormality t(4;14) were significantly younger and had an improved OS compared to others, which can be attributed to a young age and bortezomib therapy, as previously suggested. In conclusion, the R-ISS was less successful than the ISS in discriminating between stages II and III among bortezomib-treated patients with MM in an aging society, which might be attributable to the inclusion of t(4;14) in the R-ISS categorization strategy.

## INTRODUCTION

To date, numerous risk stratification systems have been developed and validated for multiple myeloma (MM). Of these, the International Staging System (ISS), which was introduced in 2005 [1], is among the most representative. The ability of this system to provide very simple but robust survival predictions in patients with MM, has been validated in many independent cohorts [2–4]. Since the introduction of the ISS, several studies have elucidated the prognostic significance of some cytogenetic abnormalities (CA) detected using interphase fluorescence *in situ* hybridization (iFISH), including del(17p), t(4;14), and t(14;16); as well as elevated serum levels of lactate dehydrogenase (LDH), in patients with MM [5–9].

In 2015, the International Myeloma Working Group developed the Revised ISS (R-ISS), which combined ISS with the status of high-risk CAs (detected by iFISH) and serum levels of LDH, to identify three MM entities with clearly different outcomes [10]. However, although MM tends to affect older adults, the original and validation studies of the R-ISS included relatively younger patients (median age: ∼65 years) [11–15]. This is of particular concern, given the rapidly aging global population in many developed Western countries. Additionally, several studies have reported that the prevalence of high-risk CAs tend to be higher among younger patients [16, 17], suggesting that the inclusion of only these patients in prognostic studies might not reflect the intrinsic prognostic value of high-risk CAs, especially in terms of overall survival (OS). Furthermore, although studies reported that bortezomib-containing therapies can reverse the unfavorable prognostic impact of t(4;14) CA [18, 19], most original and validation studies of the R-ISS included few patients who had been treated with bortezomib. Therefore, the results of these studies are less applicable to patients in the era of novel targeted agents.

Today, Japan is considered one of the most aged countries worldwide and a potential example of future situations for other developed countries. In addition, the majority of current Japanese patients with MM are treated with bortezomib. Therefore, we hypothesized that the R-ISS-based prognostication of patients with MM may be somewhat unreliable in Japan. Here, we analyzed the prognostic performance of the R-ISS using a real-world cohort of Japanese patients treated in the era of novel targeted agents.

## RESULTS

### Patients’ demographic and baseline characteristics stratified by ISS and R-ISS stages

The clinical characteristics of all patients in the cohort are summarized overall and by ISS and R-ISS stages in Table 1. The 400 enrolled patients had a median age of 72 (interquartile range [IQR]: 64–79) years. The median observation period was 37.9 (IQR: 16.4–67.9) months. Consistent with previous reports [10, 20], 39 (9.8%), 46 (11.5%), 11 (2.8%), and 91 (22.8%) patients harbored the del(17p), t(4;14), t(14;16), and any high-risk CA, respectively. There was no difference in the prevalence of each high-risk CA across the 3 hospitals (Supplementary Table 1). Ninety-eight (24.5%), 121 (30.2%), and 181 (45.2%) patients were classified as ISS stages I, II, and III, respectively, while 66 (16.5%), 243 (60.8%), and 91 (22.8%) patients were classified as R-ISS stages I, II, and III, respectively. Additionally, 384 (96.0%) patients were treated with bortezomib.

**Table 1:** Demographic and baseline clinical characteristics of patients and comparisons according to ISS and R-ISS stages

Clinical factor	All cohort	ISS				R-ISS			
	*n* = 400	Stage I	Stage II	Stage III	*P*	Stage I	Stage II	Stage III	*P*
		*n* = 98	*n* = 121	*n* = 181		*n* = 66	*n* = 243	*n* = 91	
Observation period, months [median (IQR)]	37.9 (16.4, 67.9)	45.1 (21.4, 80.1)	40.4 (18.8, 63.4)	31.2 (11.8, 56.4)	0.001	45.7 (20.5, 84.4)	37.8 (17.5, 62.3)	33.6 (11.3, 56.9)	0.016
Age, years [median (IQR)]	72 (64, 79)	68 (62, 75)	73 (66, 79)	73 (66, 81)	<0.001	68 (61, 75)	74 (66, 80)	70 (60, 77)	0.001
Sex, male (%)	207 (51.7)	50 (51.0)	53 (43.8)	104 (57.5)	0.066	36 (54.5)	124 (51.0)	47 (51.6)	0.87
Albumin, g/dL [median (IQR)]	3.5 (2.9, 4.0)	4.0 (3.7, 4.3)	3.4 (3.0, 3.8)	3.1 (2.6, 3.6)	<0.001	4.0 (3.6, 4.3)	3.4 (2.9, 4.0)	3.0 (2.6, 3.7)	<0.001
Beta 2-microglobulin, mg/L [median (IQR)]	4.3 (2.8, 7.9)	2.4 (1.9, 2.8)	3.8 (3.0, 4.5)	8.2 (6.2, 12.1)	<0.001	2.4 (1.9, 2.8)	4.3 (3.1, 7.0)	8.0 (6.0, 10.4)	<0.001
Creatinine, mg/dL [median (IQR)]	0.92 (0.71, 1.47)	0.72 (0.60, 0.82)	0.80 (0.69, 1.05)	1.48 (0.96, 2.81)	<0.001	0.71 (0.60, 0.81)	0.93 (0.72, 1.40)	1.40 (0.93, 2.29)	<0.001
Hemoglobin, g/dL [median (IQR)]	9.4 (8.2, 11.2)	11.5 (10.2, 13.3)	9.4 (8.6, 11.1)	8.6 (7.7, 9.9)	<0.001	11.5 (10.2, 13.5)	9.4 (8.3, 11.1)	8.5 (7.2, 9.6)	<0.001
LDH, high (%)	99 (24.8)	17 (17.3)	23 (19.0)	59 (32.6)	0.004	0 (0.0)	40 (16.5)	59 (64.8)	<0.001
High-risk CA (%)									
Any high-risk CA	91 (22.8)	16 (16.3)	21 (17.5)	54 (29.8)	0.009	0 (0.0)	37 (15.3)	54 (59.3)	<0.001
Del(17p)	39 (9.8)	9 (9.2)	7 (5.8)	23 (12.7)	0.13	0 (0.0)	16 (6.6)	23 (25.3)	<0.001
t(4;14)	46 (11.5)	8 (8.2)	13 (10.7)	25 (13.8)	0.35	0 (0.0)	21 (8.6)	25 (27.5)	<0.001
t(14;16)	11 (2.8)	2 (2.0)	2 (1.7)	7 (3.9)	0.45	0 (0.0)	4 (1.6)	7 (7.7)	0.004
DS, stage III (%)	258 (64.5)	39 (39.8)	60 (49.6)	159 (87.8)	<0.001	27 (40.9)	151 (62.1)	80 (87.9)	<0.001
BOR use (%)	384 (96.0)	96 (98.0)	111 (91.7)	177 (97.8)	0.016	64 (97.0)	231 (95.1)	89 (97.8)	0.47
LEN use (%)	303 (75.8)	76 (77.6)	90 (74.4)	137 (75.7)	0.99	49 (74.2)	184 (75.7)	70 (76.9)	0.92
Induction regimen (%)									
Doublet	113 (28.3)	24 (24.5)	41 (33.9)	48 (26.5)	0.24	19 (28.8)	71 (29.2)	23 (25.3)	0.77
Triplet	287 (71.8)	74 (75.5)	80 (66.1)	133 (73.5)		47 (71.2)	172 (70.8)	68 (74.7)	
ASCT recipients (%)	96 (24.0)	35 (35.7)	27 (22.3)	34 (18.8)	0.006	24 (36.4)	54 (22.2)	18 (19.8)	0.033
Outcome (%)									
Alive	220 (55.0)	69 (70.4)	69 (57.0)	82 (45.3)	<0.001	50 (75.8)	126 (51.9)	44 (48.4)	0.001
Dead	180 (45.0)	29 (29.6)	52 (43.0)	99 (54.7)		16 (24.2)	117 (48.1)	47 (51.6)	

Abbreviations: ASCT; autologous stem cell transplantation, BOR; bortezomib, CA; cytogenetic abnormality, DS; Durie–Salmon, IQR; interquartile range, ISS; International Staging System, LDH; lactate dehydrogenase, LEN; lenalidomide, R-ISS; revised International Staging System.

As previously described [21], ISS stage I included younger patients (median age: 68 years) when compared to patients with stages II and III disease, whereas these latter two stages did not differ significantly regarding age (median: both 73 years; *P =* 0.58). Accordingly, more patients were treated with autologous stem cell transplantation (ASCT) in ISS and R-ISS stage I compared with other stages. R-ISS stages II and III differed significantly in terms of age, with the former including significantly older patients than the latter (median ages: 74 and 70 years, respectively; *P =* 0.001). No significant differences in the uses of bortezomib and lenalidomide as well as in the induction regimens (doublet vs. triplet) were observed across the R-ISS stages.

### Comparison of the prognostic performances of ISS and R-ISS for OS

**Figure 1:**
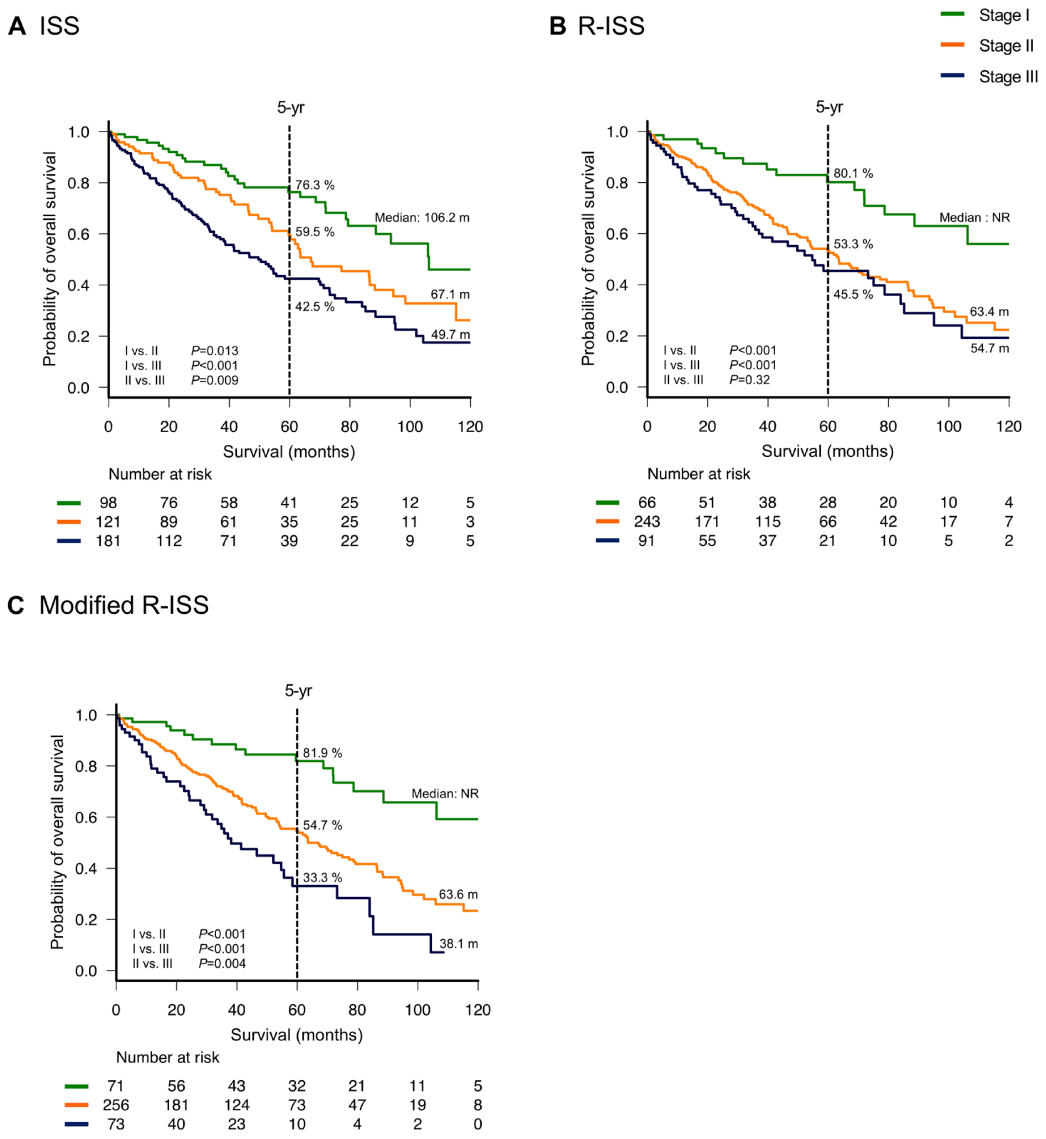
Overall survival by stage Overall survival was calculated according to the International Staging System (ISS) (**A**), revised (R)-ISS (**B**) and modified R-ISS (**C**).

The Kaplan–Meier OS curves according to the ISS and R-ISS stages are shown in Figure 1A and 1B, respectively. The three groups of patients categorized by ISS stage differed significantly in terms of survival duration (median OS: 106.2, 67.1, and 49.7 months for ISS stages I, II, and III, respectively; *P =* 0.013, <0.001, and 0.009 for ISS stage I vs. II, stage I vs. III, and stage II vs. III, respectively). In contrast, no significant differences in OS were observed between R-ISS stages II and III (median OS: 63.4 and 54.7 months, respectively; *P =* 0.32). However, patients in R-ISS stage I had a significantly longer OS (median: not reached) than those in R-ISS stages II and III (*P <* 0.001 for both R-ISS stage I vs. II and stage I vs. III). There were 131 (32.8%) patients who were censored by the end of year 5.

**Figure 2:**
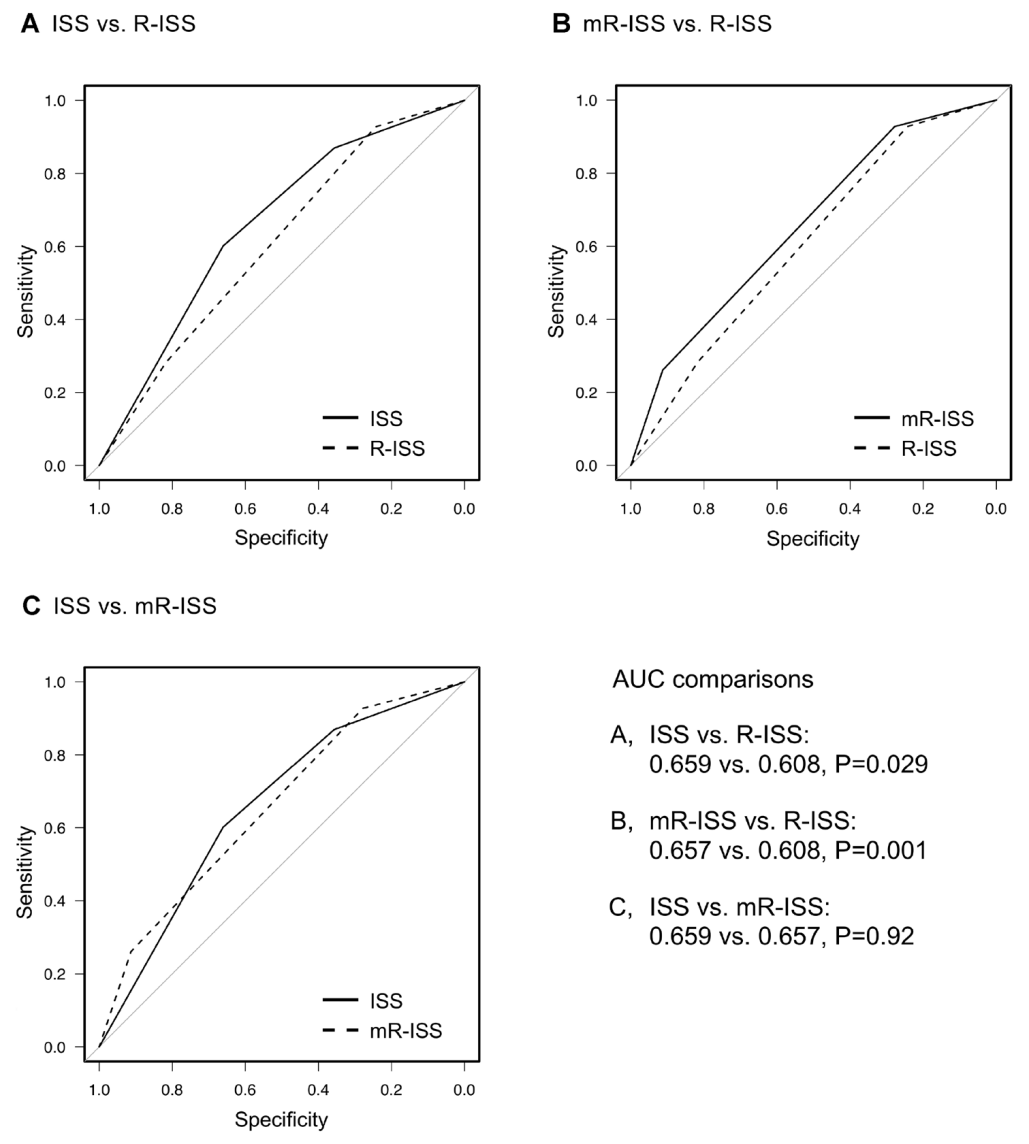
Comparisons of the area under the receiver operating characteristic (ROC) curves of each prognostic system used to predict death within 5 years International Staging System (ISS) vs. revised (R)-ISS (**A**), modified (m)R-ISS vs. R-ISS (**B**) and ISS vs. mR-ISS (**C**).

Receiver operating characteristics (ROC) curves were developed to compare the prognostic performances of the ISS and R-ISS. Notably, the area under the curve (AUC) was significantly greater for the ISS than for the R-ISS (0.659 vs. 0.608, respectively; *P =* 0.029, Figure 2A). We performed multivariate analyses for each system adjusting for age to evaluate their capability to discriminate between stage II and III. The ISS predicted a significantly poorer OS for patients with stage III compared to stage II even after adjusting for advanced age (≥70 years), whereas R-ISS failed to show significant discrimination capability in similar settings (Supplementary Table 2A and 2B).

Next, we analyzed OS according to the ISS and R-ISS stages in different age groups (younger or older than 70 years; Supplementary Figure 1). The R-ISS yielded a poor performance relative to the ISS in distinguishing OS between younger patients in stages II and III (Supplementary Figure 1A and 1B). However, no poor performance, relative to the ISS in distinguishing OS between older patients in stages II and III (Supplementary Figure 1C and 1D) was shown. No significant difference in the AUC was detected between the ISS and R-ISS among younger (0.686 vs. 0.653, respectively; *P =* 0.34) or older patients (0.592 vs. 0.549, respectively; *P =* 0.29).

### Clinical characteristics and OS among patients upgraded or downgraded during recategorization from the ISS to the R-ISS

**Figure 3:**
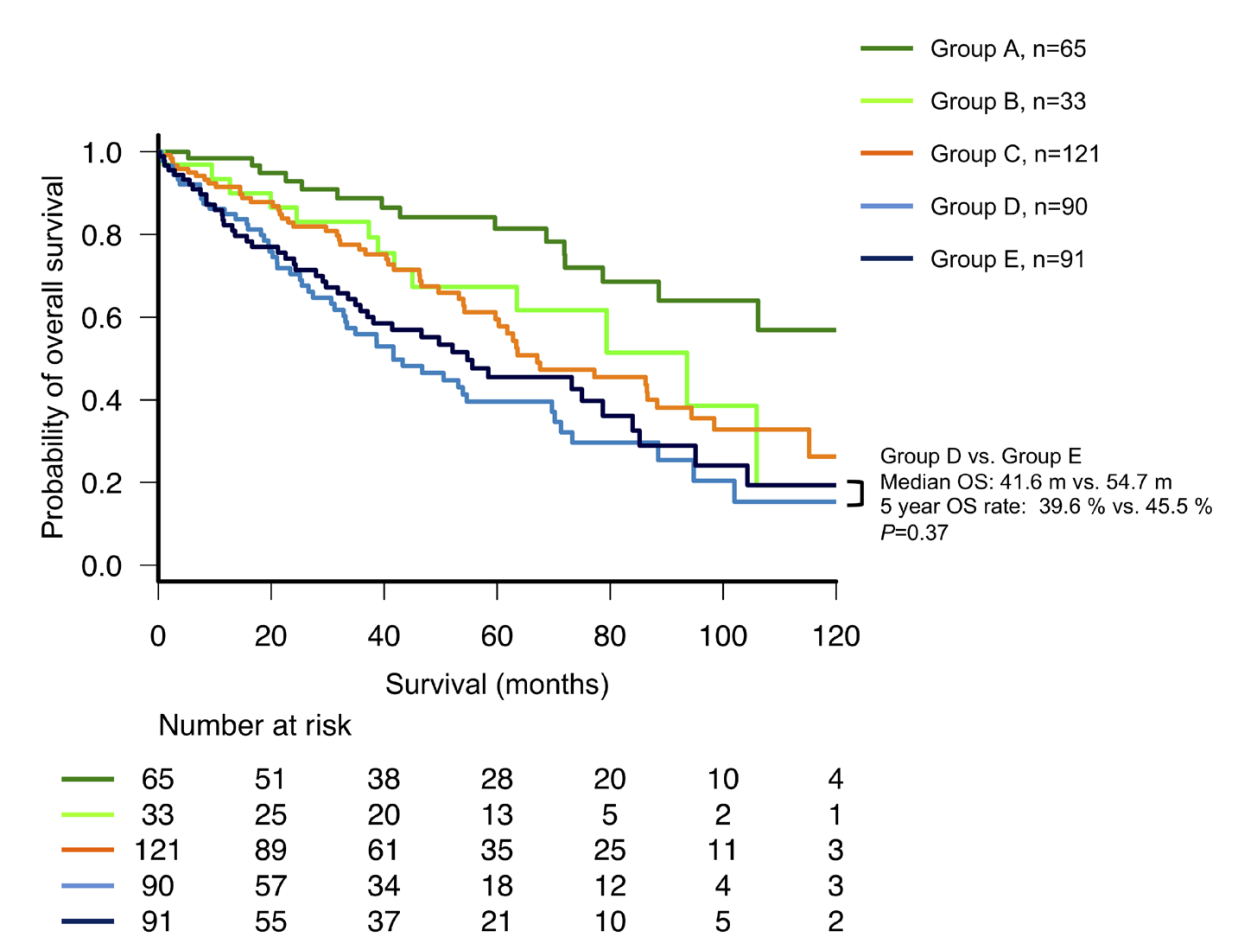
Subgroup analysis of overall survival (OS) among patients with multiple myeloma who were recategorized from the International Staging System (ISS) to the revised (R)-ISS

To identify the cause of above findings, we determined five groups of patients based on the following recategorizations: ISS stage I to R-ISS stage I (group A, 66 patients), ISS stage I to R-ISS stage II (group B, 32 patients), ISS stage II to R-ISS stage II (group C, 121 patients), ISS stage III to R-ISS stage II (group D, 90 patients), and ISS stage III to R-ISS stage III (group E, 91 patients), (Supplementary Figure 2). Although the length of OS was expected to decrease from one group to another, Group D actually had a shorter OS than Group E, although this difference was not significant (median OS: 41.6 and 54.7 months, respectively; *P =* 0.37, Figure 3). The clinical characteristics of patients in Groups D and E are shown in Supplementary Table 3. t(4;14) was the most frequently observed high-risk CA among patients in Group E, detected in approximately 30% of patients. Accordingly, patients in Group D were significantly older than those in Group E (median ages: 77 and 70 years, respectively; *P <* 0.001). However, no significant inter-group differences were observed in serum albumin, beta 2-microglobulin (B2M), and creatinine levels; hemoglobin concentration; prevalence of Durie-Salmon stage III; and therapeutic regimen.

### Age distribution and survival in patients with or without high-risk CAs and elevated LDH levels

Supplementary Table 4 presents the age distributions according to the presence of high-risk CAs and elevated LDH levels. Patients with high-risk CAs were significantly younger than those without (median: 69 and 73 years, respectively; *P <* 0.001). Specifically, patients harboring t(4;14) and t(14;16) were significantly younger than those without either CA [median ages: 68 and 73 years, respectively; *P =* 0.021 for t(4;14), and 65 and 72 years, respectively; *P =* 0.009 for t(14;16)], whereas no significant difference in age was observed between patients with and without del(17p). Considering the younger age of the patients with t(4;14), more patients in this group were treated with ASCT compared with other patients [17/46 (37.0%) vs 79/354 (22.3%) patients; *P =* 0.042]. Furthermore, patients with lower and higher LDH levels did not differ significantly in terms of age.

**Figure 4:**
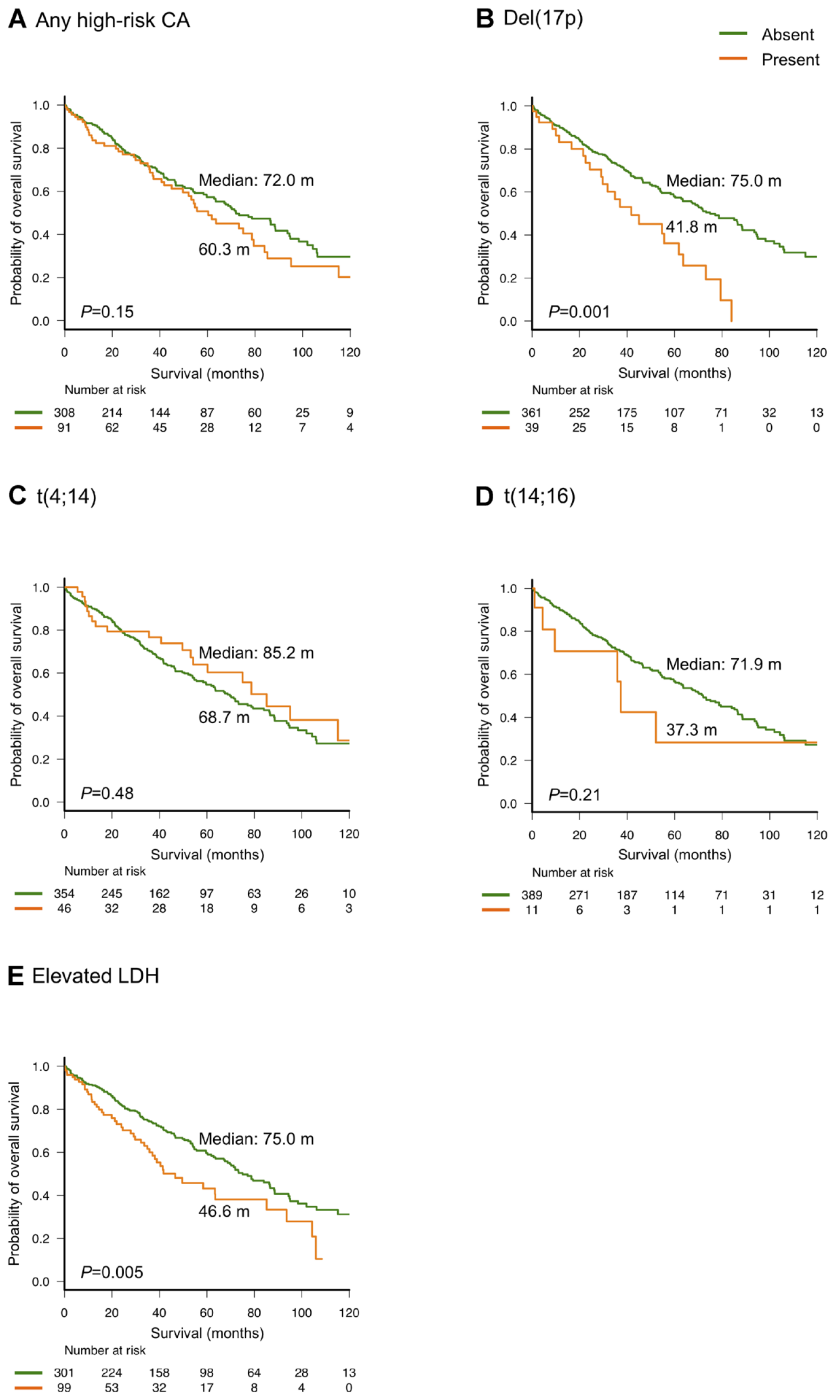
Overall survival according to high-risk cytogenetic abnormalities (CA) (**A**–**D**) and elevated lactate dehydrogenase (LDH) levels (**E**)

Figure 4 summarizes the OS outcomes according to the presence of high-risk CAs and LDH levels. Notably, OS did not differ significantly among those with any high-risk CA, relative to those without (median OS: 60.3 and 72 months, respectively; *P =* 0.15, Figure 4A); in contrast, patients with del(17p) had a significantly shorter OS, relative to those without (median OS: 41.8 and 75.0 months, respectively; *P =* 0.001, Figure 4B). OS did not differ significantly between patients with and without t(4;14) (median OS: 85.2 and 68.7 months, respectively; *P =* 0.48, Figure 4C). Patients with t(14;16) had a shorter OS than those without, although this difference was also not statistically significant (median OS: 37.3 and 71.9 months, respectively; *P =* 0.21, Figure 4D). Patients with a higher LDH level had a significantly shorter OS, compared to those with a lower LDH level (median OS: 46.6 and 75.0 months, respectively; *P =* 0.005, Figure 4E).

### Modification of the R-ISS improved the prognostication for OS

We again divided the patients into three groups using a modified R-ISS (mR-ISS) categorization in which only t(14;16) and del(17p) were included as high-risk CAs. Accordingly, the patients were categorized as follows: mR-ISS stage I included patients in ISS stage I without t(14;16) or del(17p) detected by iFISH, nor elevated LDH levels; mR-ISS stage III included patients in ISS stage III with either t(14;16) or del(17p) detected by iFISH, or elevated LDH levels; while mR-ISS stage II included patients who did not meet the criteria for mR-ISS stage I or III (Supplementary Figure 3). Accordingly, 71 (17.8%), 256 (64.0%), and 73 (18.3%) of our patients were classified as mR-ISS stages I, II, and III, respectively.

The three groups categorized by mR-ISS stage exhibited significant differences in survival (median OS: not reached, 63.6, and 38.1 months for mR-ISS stages I, II, and III, respectively; *P <* 0.001, <0.001, and 0.004 for stage I vs. II, stage I vs. III, and stage II vs. III, respectively, Figure 1C). On multivariate analysis, mR-ISS predicted a significantly poorer OS for patients with stage III compared to stage II even after adjusting for advanced age (≥70 years) (Supplementary Table 2C). We additionally developed an ROC curve for the mR-ISS, and the resulting AUC value was significantly greater than that obtained for the R-ISS (0.657 vs. 0.608, respectively; *P =* 0.001, Figure 2B). However, AUC values did not significantly differ between the ISS and mR-ISS (0.659 vs. 0.657, respectively; *P =* 0.92, Figure 2C).

## DISCUSSION

In this study, we investigated the prognostic performance of the R-ISS in a real-world context, namely, aging society patients who were treated with novel agents for symptomatic MM. As in the original study of the R-ISS, we aimed to analyze OS as the primary endpoint. Our analysis revealed a rather poor performance of the R-ISS relative to the ISS in distinguishing OS prediction between patients classified as R-ISS stages II and III. These findings may be of interest because one of the primary objectives of the R-ISS establishment was to identify patients with extremely poor prognosis as R-ISS stage III patients.

Several reliable studies have validated the utility of the R-ISS using cohorts independent from that used in the original study [11–15]. However, the patients included in these studies were uniformly younger than those in our cohort. This difference in age might partly explain why our study was the only one that failed to detect the superiority of the R-ISS relative to the ISS. Additionally, t(4;14), the most frequently occurring high-risk CA, is considered a primary genetic event in plasma cell disorders [20, 22, 23], and is known to correlate with disease progression and a younger age among patients with symptomatic MM [16, 17, 24–26]. In our real-world cohort in an aging society, the association between the performance of the R-ISS and the relatively younger age of patients with t(4;14) might have been amplified by including patients with a wide age range. Indeed, patients in R-ISS stage III more frequently presented with t(4;14), and were significantly younger than those in stage II; a result of the clear age difference between Groups D and E. However, no other component of R-ISS stage III, such as ISS stage III (vs. stage II) or an elevated LDH level, was associated with a significant age difference. Possibly, the younger ages of patients with R-ISS stage III might allow them to tolerate more intensive treatment including ASCT, which would offset the aggressiveness of the disease characterized by high-risk CAs and elevated LDH levels. Furthermore, being of a younger age per se might contribute to a favorable OS, as previously described [21, 27]. Although Group B might also have theoretically affected the prognosis of R-ISS stage II, the number of patients in this group was too small (relative to Group D) to have a significant influence on OS among patients categorized as R-ISS stage II.

As we expected, a sub-analysis of only older patients failed to yield a similar trend to that observed in the entire cohort, probably because they included few patients with t(4;14), a key subjects for our findings; suggesting that the phenomenon we found in this study necessitated the inclusion of not only older patients, but patients with a “wide age range” including both younger and older patients, as observed in real-world settings.

We further noted that almost all patients in our cohort received treatment with bortezomib, in contrast to previous studies [11–15]. Consistent with previous reports [18, 19], the present study demonstrated improved survival outcomes among patients with t(4;14); although those with other high-risk CAs had worse OS outcomes despite the use of novel agents. The increased frequency of bortezomib use and the relative youthfulness of patients harboring t(4;14) might synergistically improve OS in this population, which would undermine the performance of the R-ISS. Particularly, the impact of improved survival outcomes of these patients through the use of new therapeutic agents might be a positive point for our findings, because relatively extended OS in R-ISS stage III was observed even among the younger patients. Our additional analysis based on the mR-ISS, which was established by excluding t(4;14) as a high-risk CA, considerably improved the prognostication of OS, suggesting that t(4;14) might not be a reliable high-risk CA to be used in risk stratification systems [28]. The performance of the mR-ISS was not necessarily better than that of ISS, probably due to the excessively increased number of patients with mR-ISS stage II. It also remains questionable whether t(14;16) should be considered a high-risk CA, given the lack of reliable data regarding this rare abnormality [29]. Although t(14;16) was also observed more frequently in younger patients, similar to a previous study [16], the prognostic impact of this CA in the present study was not favorable.

We further note that our cohort included more patients in ISS stage III. These patients were considered key subjects, given the possible mechanisms described above. The particular distribution of patients in our study, which could be explained by the advanced age in our cohort [21], might have intensified the effect of categorization on the performance of the R-ISS. Accordingly, our results do not necessarily question the usefulness of the R-ISS, but rather, and more importantly, our work indicated that a careful interpretation of the R-ISS may be needed when applying this system individually to patients in real-world settings. As noted above, younger patients in R-ISS stage III who harbor t(4;14) may achieve a considerably longer OS when compared to relatively older patients in R-ISS stage II, even if both receive intensive treatment with bortezomib-containing regimens.

The present study is limited by its retrospective nature, heterogeneous treatment regimens, and relatively small sample size. In addition, it did not include details of progression-free survival along with the OS, mainly because of the unexpected discontinuation of chemotherapy in elderly patients, a frequent observation in real-world settings. Besides, the iFISH methods were not identical across the hospitals included in this study. Despite these limitations, our study findings highlight the limited usefulness of the R-ISS in the context of reasonable, naturally occurring mechanisms among patients with MM who are treated with novel agents in an aging society.

In conclusion, our study is the first to suggest that the performance of the R-ISS may be limited in discriminating OS between stage II and III when applied to real-world patients with MM who are treated with novel agents in aging populations. Furthermore, we suggest that this limitation may be attributed to the inclusion of t(4;14) as a high-risk CA in the R-ISS categorization strategy. This potential limitation suggests that the R-ISS should be carefully interpreted on an individual basis when applied to patients in a real-world setting. Our findings are of particular interest because many developed countries, including Western countries, are approaching a period of super-aging such as that observed currently in Japan. However, further studies are needed to validate our findings and develop more appropriate prognostic systems.

## METHODS

### Study design and patients

This study retrospectively analyzed the data from 400 consecutive patients who were newly diagnosed with MM and received chemotherapy between January 2006 and December 2017 at Kameda Medical Center, Chiba, Japan; Keiju Kanazawa Hospital, Ishikawa, Japan; and National Hospital Organization Okayama Medical Center, Okayama, Japan. The patients’ background and outcome data were obtained from electronic medical records, and the diagnoses and treatment responses were evaluated using the International Myeloma Working Group criteria. We included only patients who had been treated with novel agents (e.g., immunomodulatory agents or proteasome inhibitors) to reduce the prognostic impact of heterogeneity during chemotherapy. Written informed consent was obtained from all the patients or their families. The study was conducted according to the Declaration of Helsinki and was approved by the review boards of each institution.

ISS and R-ISS categorizations were performed as previously described [1, 10]. Briefly, R-ISS stage I included patients categorized as ISS stage I [a serum B2M level <3.5 mg/L and serum albumin level ≥3.5 g/dL] with neither an iFISH-detected high-risk CA [including del(17p), t(4;14), or t(14;16)], nor an elevated LDH level (above the upper limit of normal). R-ISS stage III included patients categorized as ISS stage III (a serum B2M level >5.5 mg/L) and either an iFISH-detected high-risk CA or an elevated LDH level. R-ISS stage II included all patients not classified as R-ISS stage I or III. Bone marrow samples were subjected to iFISH according to the standard methods for each institution with (Kameda Medical Center, *n* = 261) or without (Keiju Kanazawa Hospital and Okayama Medical Center, *n* = 139) CD138+ plasma cell enrichment using anti-CD138–coated magnetic MicroBeads (Miltenyi Biotech, San Diego, CA, USA). Patients were considered positive for a given CA when it was present in a percentage higher than the cutoff threshold, defined by each local laboratory. In case iFISH was performed with CD138 enrichment, the cutoff values for t(14;16), t(4;14), and del(17p) were ≥10%, ≥10%, and ≥20%, respectively [30]. In case iFISH was performed without CD138 enrichment, the cutoff values were based on the upper limit of 95% confidence interval for the expected false positive rate.

### Statistical analysis

For continuous variables, normally distributed data were presented as means and standard deviations, and non-normally distributed data were presented as medians and interquartile ranges (IQRs). The relationships of the baseline characteristics with the ISS stage, R-ISS stage, or high-risk CA status were compared using the one-way analysis of variance, Kruskal–Wallis, or chi-squared test as appropriate.

We additionally analyzed and compared the OS to elucidate the prognostic relevance of the R-ISS in an aging society. The OS durations were calculated from the date of the initial diagnosis to the date of death from any cause. The probability of OS was estimated using the Kaplan–Meier method and compared using the log-rank test. We further constructed an ROC curve to predict death within 5 years, according to each prognostic system. Patients who were alive at the last follow-up and had an observation period of <5 years were censored. Differences in the AUCs were compared using DeLong’s approach [31]. Cox proportional-hazards analyses were used to adjust for possible confounding factors. A two-tailed *P* value <0.05 was considered statistically significant. All statistical analyses were performed using R version 3.1.2 (R Foundation for Statistical Computing, Vienna, Austria).

## SUPPLEMENTARY MATERIALS FIGURES AND TABLES



## Abbreviations

ASCT: autologous stem cell transplantation
AUC: area under the curve
B2M: beta 2-microglobulin
CA: cytogenetic abnormalities
iFISH: interphase fluorescence *in situ* hybridization
IQR: interquartile range
ISS: International Staging System
LDH: lactate dehydrogenase
MM: multiple myeloma
mR-ISS: modified Revised Interna tional Staging System
OS: overall survival
R-ISS: revised International Staging System
ROC: receiver operating characteristics

## References

[1] Greipp PR, San Miguel J, Durie BG, Crowley JJ, Barlogie B, Bladé J, Boccadoro M, Child JA, Avet-Loiseau H, Kyle RA, Lahuerta JJ, Ludwig H, Morgan G (2005). International staging system for multiple myeloma. J Clin Oncol.

[2] Hungria VT, Maiolino A, Martinez G, Colleoni GW, Coelho EO, Rocha L, Nunes R, Bittencourt R, Oliveira LC, Faria RM, Pasquini R, Magalhães SM, Souza CA, International Myeloma Working Group Latin America (2008). Confirmation of the utility of the International Staging System and identification of a unique pattern of disease in Brazilian patients with multiple myeloma. Haematologica.

[3] Hari PN, Zhang MJ, Roy V, Pérez WS, Bashey A, To LB, Elfenbein G, Freytes CO, Gale RP, Gibson J, Kyle RA, Lazarus HM, McCarthy PL (2009). Is the International Staging System superior to the Durie-Salmon staging system? A comparison in multiple myeloma patients undergoing autologous transplant. Leukemia.

[4] Kastritis E, Zervas K, Symeonidis A, Terpos E, Delimbassi S, Anagnostopoulos N, Michali E, Zomas A, Katodritou E, Gika D, Pouli A, Christoulas D, Roussou M (2009). Improved survival of patients with multiple myeloma after the introduction of novel agents and the applicability of the International Staging System (ISS): an analysis of the Greek Myeloma Study Group (GMSG). Leukemia.

[5] Fonseca R, Blood E, Rue M, Harrington D, Oken MM, Kyle RA, Dewald GW, Van Ness B, Van Wier SA, Henderson KJ, Bailey RJ, Greipp PR (2003). Clinical and biologic implications of recurrent genomic aberrations in myeloma. Blood.

[6] Jaksic W, Trudel S, Chang H, Trieu Y, Qi X, Mikhael J, Reece D, Chen C, Stewart AK (2005). Clinical outcomes in t(4;14) multiple myeloma: a chemotherapy-sensitive disease characterized by rapid relapse and alkylating agent resistance. J Clin Oncol.

[7] Gutierrez NC, Castellanos MV, Martin ML, Mateos MV, Hernández JM, Fernández M, Carrera D, Rosinol L, Ribera JM, Ojanguren JM, Palomera L, Gardella S, Escoda L, GEM/PETHEMA Spanish Group (2007). Prognostic and biological implications of genetic abnormalities in multiple myeloma undergoing autologous stem cell transplantation: t(4;14) is the most relevant adverse prognostic factor, whereas RB deletion as a unique abnormality is not associated with adverse prognosis. Leukemia.

[8] Chang H, Yeung J, Qi C, Xu W (2007). Aberrant nuclear p53 protein expression detected by immunohistochemistry is associated with hemizygous P53 deletion and poor survival for multiple myeloma. Br J Haematol.

[9] Terpos E, Katodritou E, Roussou M, Pouli A, Michalis E, Delimpasi S, Parcharidou A, Kartasis Z, Zomas A, Symeonidis A, Viniou NA, Anagnostopoulos N, Economopoulos T, Greek Myeloma Study Group, Greece (2010). High serum lactate dehydrogenase adds prognostic value to the international myeloma staging system even in the era of novel agents. Eur J Haematol.

[10] Palumbo A, Avet-Loiseau H, Oliva S, Lokhorst HM, Goldschmidt H, Rosinol L, Richardson P, Caltagirone S, Lahuerta JJ, Facon T, Bringhen S, Gay F, Attal M (2015). Revised International Staging System for Multiple Myeloma: A Report From International Myeloma Working Group. J Clin Oncol.

[11] Jimenez-Zepeda VH, Duggan P, Neri P, Rashid-Kolvear F, Tay J, Bahlis NJ (2016). Revised International Staging System Applied to Real World Multiple Myeloma Patients. Clin Lymphoma Myeloma Leuk.

[12] Kastritis E, Terpos E, Roussou M, Gavriatopoulou M, Migkou M, Eleutherakis-Papaiakovou E, Fotiou D, Ziogas D, Panagiotidis I, Kafantari E, Giannouli S, Zomas A, Konstantopoulos K, Dimopoulos MA (2017). Evaluation of the Revised International Staging System in an independent cohort of unselected patients with multiple myeloma. Haematologica.

[13] Tandon N, Rajkumar SV, LaPlant B, Pettinger A, Lacy MQ, Dispenzieri A, Buadi FK, Gertz MA, Hayman SR, Leung N, Go RS, Dingli D, Kapoor P (2017). Clinical utility of the Revised International Staging System in unselected patients with newly diagnosed and relapsed multiple myeloma. Blood Cancer J.

[14] Cho H, Yoon DH, Lee JB, Kim SY, Moon JH, Do YR, Lee JH, Park Y, Lee HS, Eom HS, Shin HJ, Min CK, Kim JS, the Korean Multiple Myeloma Working Party (2017). Comprehensive evaluation of the revised international staging system in multiple myeloma patients treated with novel agents as a primary therapy. Am J Hematol.

[15] Walker I, Coady A, Neat M, Ladon D, Benjamin R, El-Najjar I, Kazmi M, Schey S, Streetly M (2018). Is the revised International staging system for myeloma valid in a real world population?. Br J Haematol.

[16] Ross FM, Ibrahim AH, Vilain-Holmes A, Winfield MO, Chiecchio L, Protheroe RK, Strike P, Gunasekera JL, Jones A, Harrison CJ, Morgan GJ, Cross NC, UK Myeloma Forum (2005). Age has a profound effect on the incidence and significance of chromosome abnormalities in myeloma. Leukemia.

[17] Avet-Loiseau H, Hulin C, Campion L, Rodon P, Marit G, Attal M, Royer B, Dib M, Voillat L, Bouscary D, Caillot D, Wetterwald M, Pegourie B (2013). Chromosomal abnormalities are major prognostic factors in elderly patients with multiple myeloma: the intergroupe francophone du myelome experience. J Clin Oncol.

[18] San Miguel JF, Schlag R, Khuageva NK, Dimopoulos MA, Shpilberg O, Kropff M, Spicka I, Petrucci MT, Palumbo A, Samoilova OS, Dmoszynska A, Abdulkadyrov KM, Schots R, VISTA Trial Investigators (2008). Bortezomib plus melphalan and prednisone for initial treatment of multiple myeloma. N Engl J Med.

[19] Avet-Loiseau H, Leleu X, Roussel M, Moreau P, Guerin-Charbonnel C, Caillot D, Marit G, Benboubker L, Voillat L, Mathiot C, Kolb B, Macro M, Campion L (2010). Bortezomib plus dexamethasone induction improves outcome of patients with t(4;14) myeloma but not outcome of patients with del(17p). J Clin Oncol.

[20] Kumar SK, Rajkumar V, Kyle RA, van Duin M, Sonneveld P, Mateos MV, Gay F, Anderson KC (2017). Multiple myeloma. Nat Rev Dis Primers.

[21] Ludwig H, Durie BG, Bolejack V, Turesson I, Kyle RA, Blade J, Fonseca R, Dimopoulos M, Shimizu K, San Miguel J, Westin J, Harousseau JL, Beksac M (2008). Myeloma in patients younger than age 50 years presents with more favorable features and shows better survival: an analysis of 10 549 patients from the International Myeloma Working Group. Blood.

[22] Dewald GW, Therneau T, Larson D, Lee YK, Fink S, Smoley S, Paternoster S, Adeyinka A, Ketterling R, Van Dyke DL, Fonseca R, Kyle R (2005). Relationship of patient survival and chromosome anomalies detected in metaphase and/or interphase cells at diagnosis of myeloma. Blood.

[23] Lauring J, Abukhdeir AM, Konishi H, Garay JP, Gustin JP, Wang Q, Arceci RJ, Matsui W, Park BH (2008). The multiple myeloma associated MMSET gene contributes to cellular adhesion, clonogenic growth, and tumorigenicity. Blood.

[24] Brito JL, Walker B, Jenner M, Dickens NJ, Brown NJ, Ross FM, Avramidou A, Irving JA, Gonzalez D, Davies FE, Morgan GJ (2009). MMSET deregulation affects cell cycle progression and adhesion regulons in t(4;14) myeloma plasma cells. Haematologica.

[25] Lopez-Corral L, Gutiérrez NC, Vidriales MB, Mateos MV, Rasillo A, Garcia-Sanz R, Paiva B, San Miguel JF (2011). The progression from MGUS to smoldering myeloma and eventually to multiple myeloma involves a clonal expansion of genetically abnormal plasma cells. Clin Cancer Res.

[26] Rajkumar SV, Gupta V, Fonseca R, Dispenzieri A, Gonsalves WI, Larson D, Ketterling RP, Lust JA, Kyle RA, Kumar SK (2013). Impact of primary molecular cytogenetic abnormalities and risk of progression in smoldering multiple myeloma. Leukemia.

[27] Ludwig H, Bolejack V, Crowley J, Bladé J, Miguel JS, Kyle RA, Rajkumar SV, Shimizu K, Turesson I, Westin J, Sonneveld P, Cavo M, Boccadoro M (2010). Survival and years of life lost in different age cohorts of patients with multiple myeloma. J Clin Oncol.

[28] Mikhael JR, Dingli D, Roy V, Reeder CB, Buadi FK, Hayman SR, Dispenzieri A, Fonseca R, Sher T, Kyle RA, Lin Y, Russell SJ, Kumar S, Mayo Clinic (2013). Management of newly diagnosed symptomatic multiple myeloma: updated Mayo Stratification of Myeloma and Risk-Adapted Therapy (mSMART) consensus guidelines 2013. Mayo Clin Proc.

[29] Avet-Loiseau H, Malard F, Campion L, Magrangeas F, Sebban C, Lioure B, Decaux O, Lamy T, Legros L, Fuzibet JG, Michallet M, Corront B, Lenain P, Intergroupe Francophone du Myélome (2011). Translocation t(14;16) and multiple myeloma: is it really an independent prognostic factor?. Blood.

[30] Ross FM, Avet-Loiseau H, Ameye G, Gutiérrez NC, Liebisch P, O’Connor S, Dalva K, Fabris S, Testi AM, Jarosova M, Hodkinson C, Collin A, Kerndrup G, European Myeloma Network (2012). Report from the European Myeloma Network on interphase FISH in multiple myeloma and related disorders. Haematologica.

[31] DeLong ER, DeLong DM, Clarke-Pearson DL (1988). Comparing the areas under two or more correlated receiver operating characteristic curves: a nonparametric approach. Biometrics.

