# Supplementary material for: Improved survival outcomes and relative youthfulness of multiple myeloma patients with t(4;14) receiving novel agents are associated with poorer performance of the revised international staging system in a real aging society
Source: Oncotarget. 2019 Jan 15;10(5):595–605. doi: 10.18632/oncotarget.26562 (PMC6355174; doi:10.18632/oncotarget.26562)
Supplement: Supplementary file 1 [file oncotarget-10-595-s001.pdf]

Improved survival outcomes and relative youthfulness of multiple myeloma patients with t(4;14) receiving novel agents are associated with poorer performance of the revised international staging system in a real aging society

SUPPLEMENTARY MATERIALS

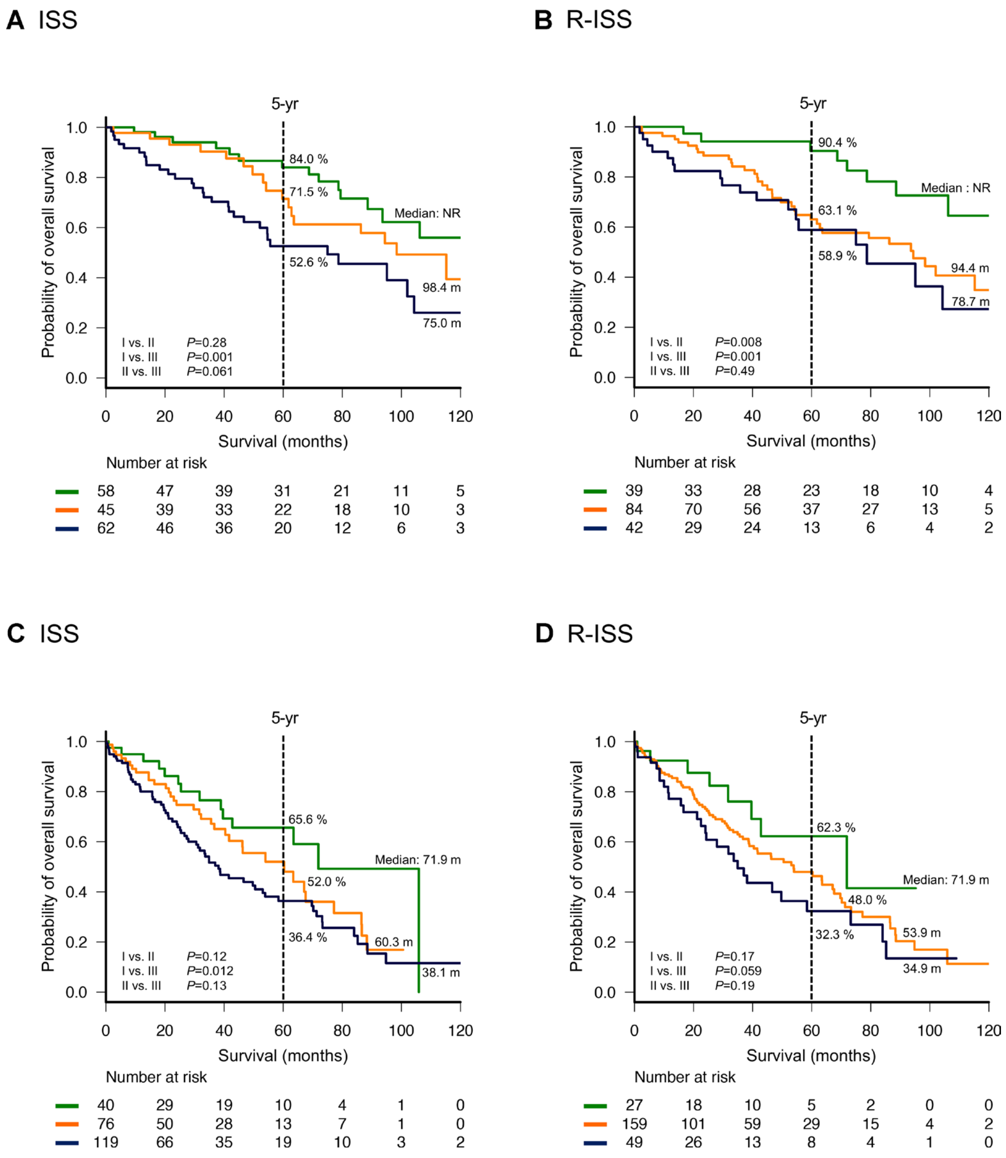

Supplementary Figure 1: Overall survival according to the International Staging System (ISS) and R-ISS stages in different age groups. The data were stratified by younger (**A** and **B**) or older (**C** and **D**) than 70 years.

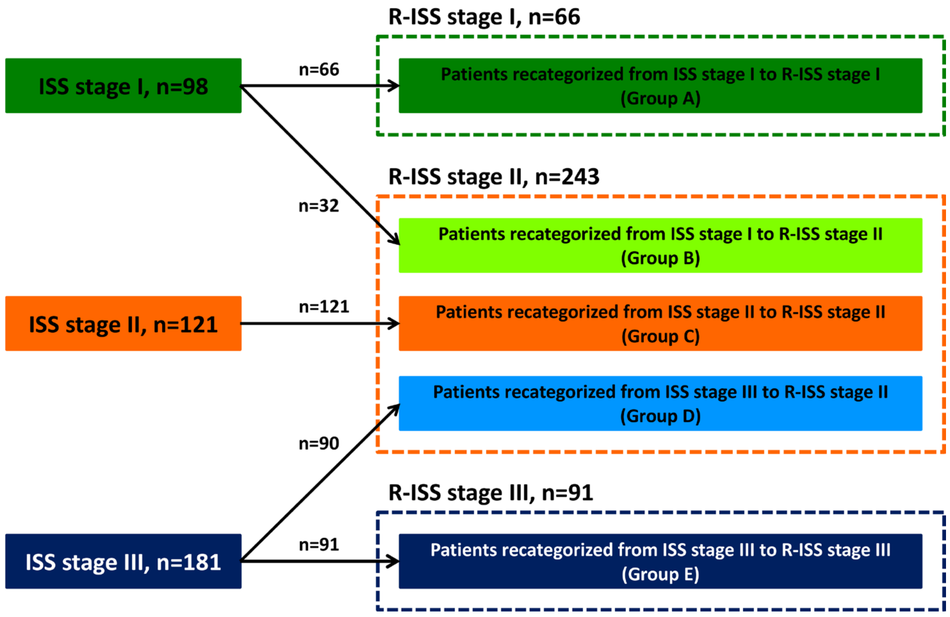

Supplementary Figure 2: Recategorization of patients with multiple myeloma (MM) from International Staging System (ISS) stages to revised (R)-ISS stages.

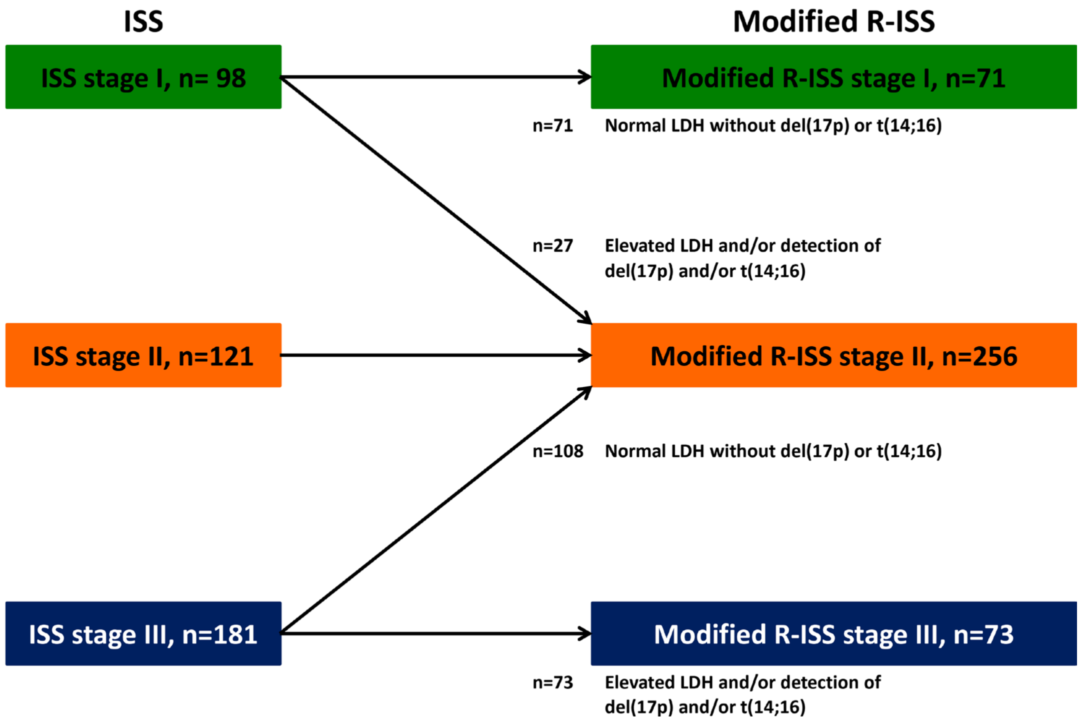

Supplementary Figure 3: Restaging of patients according to a modification of the revised International Staging System (R-ISS). We combined the standard ISS categories with lactate dehydrogenase (LDH) levels and the high-risk cytogenetic abnormalities (CA) del(17p) and/or t(14;16).

Supplementary Table 1: Comparison of the distribution of each high-risk cytogenetic abnormality with previous reports and across the enrolled hospitals

	General distribution, (%) [1–5]	Kameda Medical Center (*n* = 261), *n* (%)	Keiju Kanazawa Hospital (*n* = 79), *n* (%)	Okayama Medical Center (*n* = 60), *n* (%)	*P*
Any high-risk CA	(20–25)	60 (23.0)	19 (24.1)	12 (20.0)	0.84
Del(17p)	(5–10)	26 (10.0)	8 (10.1)	5 (8.3)	0.92
t(4;14)	(10–20)	29 (11.1)	11 (13.9)	6 (10.0)	0.73
t(14;16)	(2–3)	5 (1.9)	3 (3.8)	3 (5.0)	0.34

Abbreviation: CA; cytogenetic abnormality.

SUPPLEMENTARY REFERENCES

1. Xiong W, Wu X, Starnes S, Johnson SK, Haessler J, Wang S, Chen L, Barlogie B, Shaughnessy JD Jr, Zhan F. An analysis of the clinical and biologic significance of TP53 loss and the identification of potential novel transcriptional targets of TP53 in multiple myeloma. Blood. 2008; 112:4235–46. https://doi.org/10.1182/blood-2007-10-119123.
2. Palumbo A, Avet-Loiseau H, Oliva S, Lokhorst HM, Goldschmidt H, Rosinol L, Richardson P, Caltagirone S, Lahuerta JJ, Facon T, Bringhen S, Gay F, Attal M, et al. Revised International Staging System for Multiple Myeloma: A Report From International Myeloma Working Group. J Clin Oncol. 2015; 33:2863–9. https://doi.org/10.1200/JCO.2015.61.2267.
3. Kumar SK, Rajkumar V, Kyle RA, van Duin M, Sonneveld P, Mateos MV, Gay F, Anderson KC. Multiple myeloma. Nat Rev Dis Primers. 2017; 3:17046. https://doi.org/10.1038/nrdp.2017.46.
4. Avet-Loiseau H, Leleu X, Roussel M, Moreau P, Guerin-Charbonnel C, Caillot D, Marit G, Benboubker L, Voillat L, Mathiot C, Kolb B, Macro M, Campion L, et al. Bortezomib plus dexamethasone induction improves outcome of patients with t(4;14) myeloma but not outcome of patients with del(17p). J Clin Oncol. 2010; 28: 4630–4. https://doi.org/10.1200/JCO.2010.28.3945.
5. Avet-Loiseau H, Attal M, Campion L, Caillot D, Hulin C, Marit G, Stoppa AM, Voillat L, Wetterwald M, Pegourie B, Voog E, Tiab M, Banos A, et al. Long-term analysis of the IFM 99 trials for myeloma: cytogenetic abnormalities [t(4;14), del(17p), 1q gains] play a major role in defining long-term survival. J Clin Oncol. 2012; 30:1949–52. https://doi.org/10.1200/JCO.2011.36.5726.

Supplementary Table 2: Multivariate analysis for each staging system: (A) International Staging System (ISS), (B) Revised International Staging System (R-ISS), and (C) modified Revised International Staging System (mR-ISS) evaluating the capability to discriminate overall survival between stages II and III

A. ISS

Variables	Hazard ratio	(95% Confidence interval)	*P*
Age, ≥ 70 years	2.10	(1.47–3.00)	<0.001
ISS			
Stage II	1 (Reference)		
Stage III	1.48	(1.05–2.07)	0.024

Abbreviation: ISS; International Staging System.

B. R-ISS

Variables	Hazard ratio	(95% Confidence interval)	*P*
Age, ≥ 70 years	2.16	(1.54–3.03)	<0.001
R-ISS			
Stage II	1 (Reference)		
Stage III	1.29	(0.92–1.82)	0.14

Abbreviation: R-ISS; Revised International Staging System.

C. mR-ISS

Variables	Hazard ratio	(95% Confidence interval)	*P*
Age, ≥ 70 years	2.15	(1.54–3.01)	<0.001
mR-ISS			
Stage II	1 (Reference)		
Stage III	1.82	(1.26–2.61)	0.001

Abbreviation: mR-ISS; modified Revised International Staging System.

Supplementary Table 3: Comparison of clinical characteristics of patients in Groups D and E

Clinical factors	Group D	Group E	*P*
	***n* = 90**	***n* = 91**	
Observation period, months [median (IQR)]	27.6 (13.7, 54.6)	33.6 (11.3, 56.9)	0.92
Age, years [median (IQR)]	77 (71, 82)	70 (60, 77)	<0.001
Sex, male (%)	57 (63.3)	47 (51.6)	0.13
Albumin, g/dL [median (IQR)]	3.1 (2.7, 3.5)	3.0 (2.6, 3.7)	0.69
β2-micloglobulin, mg/L [median (IQR)]	8.3 (6.4, 13.2)	8.0 (6.0, 10.4)	0.50
Creatinin, mg/dL [median (IQR)]	1.70 (0.97, 3.70)	1.40 (0.93, 2.29)	0.17
Hemoglobin, g/dL [median (IQR)]	8.8 (7.9, 10.1)	8.5 (7.2, 9.6)	0.092
LDH, high (%)	0 (0.0)	59 (64.8)	<0.001
High-risk CA (%)	0 (0.0)	54 (59.3)	<0.001
Del(17p)	0 (0.0)	23 (25.3)	<0.001
t(4;14)	0 (0.0)	25 (27.5)	<0.001
t(14;16)	0 (0.0)	7 (7.7)	0.014
DS, stage III (%)	79 (87.8)	80 (87.9)	1.0
BOR use in whole treatment (%)	88 (97.8)	89 (97.8)	1.0
LEN use in whole treatment (%)	67 (74.4)	70 (76.9)	0.73
Triplet induction chemotherapy (%)	65 (72.2)	68 (74.7)	0.73
ASCT recipients (%)	16 (17.8)	18 (19.8)	0.84
Outcome (%)			
Alive	38 (42.2)	44 (48.4)	0.45
Dead	52 (57.8)	47 (51.6)	

Abbreviations: ASCT; autologous stem cell transplantation, BOR; bortezomib, CA; cytogenetic abnormality, DS; Durie–Salmon, IQR; interquartile range, LDH; lactate dehydrogenase, LEN; lenalidomide.

Supplementary Table 4: Comparison of age in patients with or without high-risk cytogenetic abnormalities, elevated LDH levels and ISS stage III

Clinical factors	Age, years [median (IQR)]	*P*
Any high-risk CA		
Absent	73 (65, 79)	<0.001
Present	69 (59, 74)	
Del(17p)		
Absent	72 (64, 79)	0.095
Present	69 (59, 76)	
t(4;14)		
Absent	73 (64, 79)	0.021
Present	68 (59, 75)	
t(14;16)		
Absent	72 (64, 79)	0.009
Present	65 (57, 69)	
Elevated LDH		
Absent	72 (63, 78)	0.43
Present	72 (65, 78)	
ISS stage II vs. III		
Stage II	73 (66, 79)	0.58
Stage III	73 (66, 81)	

Abbreviations: CA; cytogenetic abnormality, IQR; interquartile range, ISS; International Staging System, LDH; lactate dehydrogenase.
